# Electrochemical Commodity Polymer Up‐ and Re‐Cycling: Toward Sustainable and Circular Plastic Treatment

**DOI:** 10.1002/marc.202500143

**Published:** 2025-04-18

**Authors:** Maxime Hourtoule, Sven Trienes, Lutz Ackermann

**Affiliations:** ^1^ Wöhler Research Institute for Sustainable Chemistry (WISCh) Georg‐August‐Universität Göttingen Tammannstraße 2 37077 Göttingen Germany

**Keywords:** electrocatalysis, electrochemistry, plastic recycling, polymer degradation, polymer functionalization

## Abstract

The demand for commodity plastics reaches unprecedented dimensions. In contrast to the well‐developed plethora of methods for polymer synthesis, sustainable strategies for the end‐of‐life management of plastics continue to be scarce. While mechanical re‐cycling often results in downgraded materials, chemical re‐cycling or up‐cycling offers tremendous potential for an efficient and green approach, thereby addressing the precarious treatment of post‐use plastics within a circular carbon economy. Recently, electrochemistry surfaced as a uniquely powerful tool for polymer up‐cycling via polymer functionalization or degradation obtaining either novel polymers with valorized properties or high‐value recycled small molecules, respectively. While discussing recent progress in that domain, future perspectives of electrochemical polymer modifications until January 2025 are outlined herein.

## Graphical abstract

The annual plastic production rate is continuously growing. Thus, appropriate end‐of‐life management is essential for a green future. Electrochemistry offers a unique potential for a sustainable and efficient method for the treatment of plastic waste through polymer up‐ and re‐cycling yielding high‐value small molecules or novel polymeric material usually hard to access via common polymerization techniques.

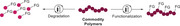

## 1 Introduction

Plastics are fantastic and omnipresent in daily life, while their versatility and high robustness render them indispensable. Their high relevance is represented by the continuously increasing annual production rate of 460 million metric tons in 2019 which is expected to grow up to more than 1014 million metric tons in 2060.^[^ ^1^ ^]^ Despite major progress in developing new materials featuring interesting properties and almost limitless applications,^[^ ^2^, ^3^, ^4^, ^5^ ^]^ their lifetime is limited, making an appropriate end‐of‐life management inevitable.^[^ ^6^ ^]^

**Scheme 1:**
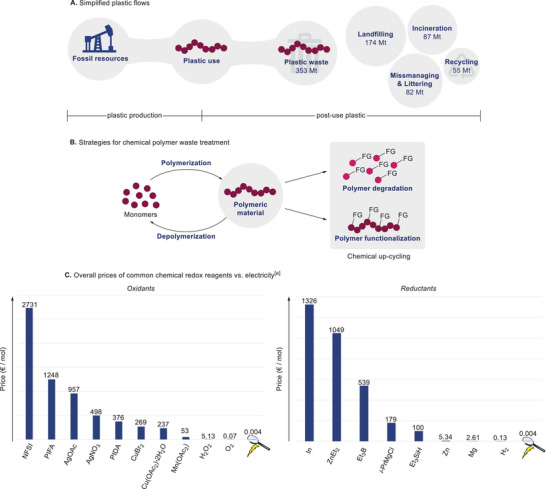
A) Simplified life‐cycle of plastic from production to post‐use strategies. B) Methods for chemical polymer waste treatment through re‐cycling. C) Comparison of costs for common chemical oxidants and reductants versus electricity in € per mol. [a] Average prices for the largest units obtained from ABCR, Fisher Scientific, Sigma‐Aldrich, and Nippon Gases (for H_2_ and O_2_). FG = functional group.

Considering current strategies for the sourcing of base chemicals for plastic production, fabrication as well as for their disposal, lifetime plastic flows are rather one‐sided. In this context, plastics are predominantly originated from fossil‐based feedstocks^[^ ^7^ ^]^ and often end up being landfilled or incinerated (**Scheme** **1**A).^[^ ^8^, ^9^, ^10^ ^]^ In sharp contrast, only 55 million tons of the 353 million tons annually generated plastic waste were collected for re‐cycling; with ultimately 9% of actually recycled waste, being far away from an ideal circular carbon economy.^[^ ^1^ ^]^ Overall, the total life‐cycle assessment and carbon footprint analysis are associated with significant detrimental environmental influences. A vast majority of plastic originated from fossil resources and plastic production is typically a high‐energy process causing tremendous greenhouse gas emission as well as the incineration of post‐use plastic.^[^ ^1^, ^11^ ^]^ Furthermore, mismanaged, non‐incinerated plastic waste represents a threat to the environment and wildlife through uncontrolled micro‐ and macroplastics leakage. In 2019, 22 million tons of plastic waste leaked into the environment, thereby polluting marine and terrestrial ecosystems. Due to their low biodegradability, plastics continuously accumulate in the environment and leach hazardous chemicals and additives with potentially significant consequences, ultimately affecting the human health via the food chain.^[^ ^1^ ^]^

Given all these negative aspects of current plastic management, sustainable and efficient methods for the up‐cycling and re‐cycling of plastics are of the highest societal demand. Current strategies for a circular plastic management are based on two fundamental strategies:^[^ ^12^, ^13^ ^]^ First, mechanical re‐cycling, the predominantly applied re‐cycling pathway, comprises sorting, cleaning, shredding, and subsequent melting of plastic waste, often resulting in plastic pellets or other polymeric products.^[^ ^14^ ^]^ However, mechanical re‐cycling is not applicable to all polymeric materials and is often associated with a significant modification of the properties of the original material, thus resulting in a downgraded material.^[^ ^15^ ^]^ In sharp contrast, chemical re‐cycling as the second strategy offers the unique potential of either recovering the monomeric building blocks through depolymerization strategy, or to obtain new, high‐value materials through chemical up‐cycling (Scheme 1B). Thus, chemical re‐cycling of plastic waste, an attractive feedstock due to its high abundance and low cost of material, potentially represents an ideal approach to enable an expedient treatment of post‐consumer materials. However, thus far, chemical re‐cycling represents only a niche in the end‐of‐life plastic treatment as less than 1% of the global plastic production in 2021 ended in this process.^[^ ^15^, ^16^ ^]^ Despite this low percentage, this method bears huge potential as a sustainable and seminal technology for plastic waste treatment, which will be elaborated in detail within this Perspective. While methods for chemical polymer up‐cycling have been studied by means of thermal^[^ ^17^, ^18^, ^19^, ^20^, ^21^, ^22^, ^23^, ^24^, ^25^, ^26^, ^27^, ^28^ ^]^ or photocatalytic^[^ ^25^, ^29^, ^30^, ^31^, ^32^, ^33^, ^34^, ^35^, ^36^, ^37^, ^38^, ^39^, ^40^ ^]^ approaches, electrochemical polymer functionalization and degradation are less explored and gained considerable momentum recently.^[^ ^25^, ^36^, ^41^, ^42^, ^43^, ^44^, ^45^ ^]^ Especially the use of electrochemistry, as a traceless, sustainable, and easily tunable redox agent offers unique opportunities in the modification and degradation of polymers. Additionally, and from an economical viewpoint, employing electricity as redox equivalent appears to be highly attractive comparing the price of electricity with commonly used chemical redox reagents (Scheme 1C). Current progress in this field until January 2025 and an outlook will be presented herein.

## 2 Electrochemical Polymer Up‐Cycling

While the use of electrochemistry has been established as a highly versatile and powerful tool for organic and inorganic transformations of small molecules,^[^ ^46^, ^47^, ^48^, ^49^, ^50^, ^51^ ^]^ the application of current in the domain of polymer up‐cycling and post‐use functionalization offers outstanding potential.^[^ ^43^ ^]^

### 2.1 Transition Metal‐Free, Electrochemical Polymer Modification

**Scheme 2:**
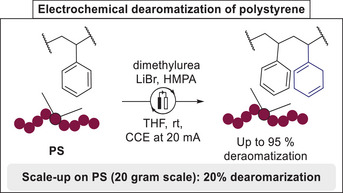
Transition metal‐free electrochemical dearomatization of polystyrene. CCE = constant current electrolysis.

In the context of polymer up‐cycling, an electrochemical dearomatization of arenes was applied to polystyrene polymers and copolymers by Sarlah.^[^ ^52^ ^]^ The standard procedure for Birch reduction appears to be inefficient to perform the dearomatization of polystyrene (PS), whereas electroreduction of arenes^[^ ^53^ ^]^ allowed a mild and effective access to reduced materials, without any alteration of average molecular weight. Moreover, this dearomatization strategy could be conducted on larger scale (up to 20 g) without cleavage of the polymer backbone, albeit with reduced efficacy. This approach has been applied to different plastic wastes and PS‐based copolymers and enabled further functionalization of the olefinic products to access new types of materials (**Scheme** **2**).

Electrochemical transformations allow mild and efficient access to functionalized polymers, that are impossible to access by direct polymerization, hence, allowing cost‐efficient and scalable access to higher value polymers.

### 2.2 Electrochemical Modification of Functionalized Polymers Employing Transition Metals

**Scheme 3:**
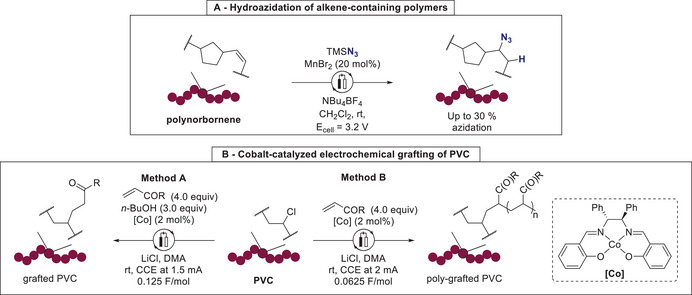
Electrochemical approaches for polymer modification employing transition metal catalysts. A. Hydroazidation of alkene‐containing polymers. B. Cobalt‐catalyzed electrochemical grafting of PVC.

The versatility and unique potential of transition metals has also been explored in the context of electrochemical polymer functionalization reactions. In 2022, Brantley described an electricity‐induced hydroazidation of alkene‐containing polymers, such as polynorbornene, in the presence of a large excess of trimethylsilyl azide.^[^ ^54^ ^]^ Azidated polymers were obtained with up to 30% azidation yield. The utility of this polymer azidation was demonstrated by further functionalization with copper‐catalyzed click reaction. However, significant chain cleavage occurred during this electrochemical hydroazidation process, hence limiting its applicability (**Scheme** **3**A). One year later, Botte demonstrated the considerable influence of 3d transition metals, such as copper or nickel, present as electrolyte and electrode material, on the electrochemical oxidation and functionalization of low‐density polyethylene (PE).^[^ ^55^ ^]^ An approach for the electrochemical up‐cycling, transition metal‐catalyzed polymer functionalization, reported by Sevov, focused on the modification of polyvinyl chloride (PVC) and polychlorinated wastes.^[^ ^56^ ^]^ Here, C–Cl bonds were selectively replaced by functionalities mimicking PVC plasticizers via electroreductive alkylation (Scheme 3B). This grafting strategy for plasticizing additives employed a cobalt salen catalyst. Interestingly, the degree of functionalization was easily controlled by the redox capacity applied during the electrolysis furnishing either grafted or poly‐grafted PVC products. Hence, the thus obtained modified novel polymeric materials exhibited distinct properties compared to the original PVC.^[^ ^56^ ^]^

### 2.3 Electrochemical Transition Metal‐Catalyzed Commodity Polymer Up‐Cycling via C–H Functionalization

**Scheme 4:**
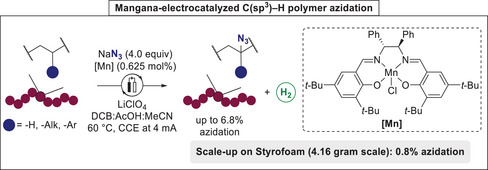
Manganaelectro‐catalyzed azidation of commodity polymers via C─H functionalization.

The functionalization of C–H bonds arguably represents the most efficient and direct way to a given target structure. Considering the high abundance of non‐functionalized polymers in plastics with ubiquitous C–H bonds, this approach is extremely attractive in the context of polymer up‐ and re‐cycling. To that extend, in 2023, Ackermann reported on the first mangana‐electrocatalytic C–H azidation of a broad range of polymers, such as PS, poly(2‐vinylnaphthalene), PS‐containing block copolymers, and especially challenging polymers like PE and polypropylene (PP) (**Scheme** **4**).^[^ ^57^ ^]^ A well‐defined manganese(III) catalyst allowed for the highly efficient and selective azidation of the corresponding polymer backbones. While the azidation proceeded smoothly with a high level of functionalization, it is noteworthy that the electrocatalytic process featured a cathodic hydrogen evolution reaction (HER), generating useful and green hydrogen as the sole byproduct. Moreover, this electrochemical C–H azidation could be carried out on larger scale of Styrofoam (4.16 g) to yield up‐cycled waste with 0.8 mol% of azidation. The post‐synthetic derivatization of the azide‐containing PS toward novel polymeric materials with altered properties further highlighted the huge potential of polymer up‐cycling as a valuable re‐cycling process.^[^ ^57^ ^]^

## 3 Electrochemical Depolymerization

The beneficial effect of electrochemistry for polymer functionalization was recently described (vide supra). However, some of these approaches resulted in a significant reduction of molecular weight that could be observed during the evaluation of suitable reaction conditions.^[^ ^54^, ^57^ ^]^ Electrochemistry thus offers the opportunity for selective and efficient polymer degradation toward high‐value small molecules.

### 3.1 Electricity‐Induced Depolymerization by Direct Electron‐Transfer

**Scheme 5:**
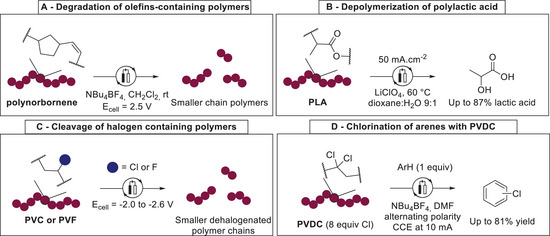
Examples for electrochemical depolymerization by direct electron‐transfer. A) Degradation of olefins‐containing polymers. B) Depolymerization of polylactic acid. C) Electrochemical cleavage of halogen containing polymers. D) Chlorination of arenes with PVDC.

Brantley described a chemoselective chain‐cleavage of alkene‐containing polynorbornene polymer (**Scheme** **5**A).^[^ ^54^ ^]^ A direct oxidation of the internal unactivated alkene of the polymer induced chain scission within the polymer chain. However, interchained radical recombination also occurred under these conditions. This method was further applied to different unsaturated polymers such as 1,4‐*cis*‐polyisoprene or poly‐(cyclooctene). Moreover, polymers with a saturated backbone, such as PS, were not affected by this depolymerization method, showcasing the chemoselectivity of this electrochemical approach. Two years later, an electrochemical depolymerization of polylactic acid (PLA) was reported by Palkovits (Scheme 5B).^[^ ^58^ ^]^ A combination of single electron‐transfer to carboxyl group and cathodic hydroxide formation allowed the efficient depolymerization of PLA to lactic acid with up to 87% yield. Halogen‐containing polymers, such as PVC or polyvinyl fluoride (PVF), were shown to be prone to selective cathodic carbon‐halogen bond reduction, hence inducing chain cleavage within the polymer backbones.^[^ ^59^, ^60^ ^]^ These methods allowed partial deconstruction of PVC and PVF polymers (Scheme 5C). Recently, Samanta reported a direct reduction of PVDC (polyvinylidene chloride), serving as a chlorine source, to achieve the chlorination of arenes or heteroarenes that could be adapted and scaled up in flow. The method achieved efficient dechlorination of PVDC (up to 98% dechlorination) and was used to access high value chlorinated arenes and heteroarenes from waste chlorine‐containing polymers (Scheme 5D).^[^ ^61^ ^]^ As shown, electrochemistry is a powerful method to selectively perform direct electron‐transfers into polymer backbones, hence inducing chain cleavage. However, this strategy is so far limited to functionalized polymer chains, hence rendering it challenging to be applied for the degradation of commodity polymers with low inherent reactivity, such as more exciting, yet more challenging PE, PP or PS.

### 3.2 Redox‐Mediator Induced Electrochemical Degradation of Diverse Polymers

**Scheme 6:**
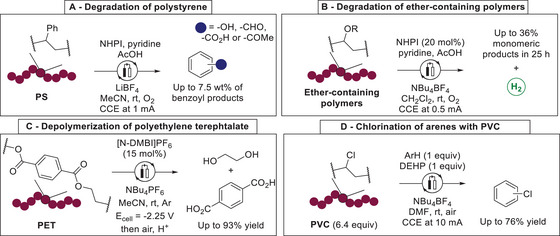
Examples for redox‐mediator induced electrochemical degradation of polymers. A) Degradation of polystyrene. B) Degradation of ether‐containing polymers. C) Depolymerization of polyethylene terephthalate. D) Chlorination of arenes with PVC.

The use of redox‐mediators to perform electron‐transfer to the polymer backbone has been more intensively investigated in the last few years. Román‐Leshkov utilized *N*‐hydroxyphtalimide (NHPI) as redox‐mediator to activate the benzylic position in PS. Under an oxygen atmosphere and constant current electrolysis (CCE), C–C bond cleavage occurred to afford up to 7.5wt% of monomeric products (**Scheme** **6**A).^[^ ^62^ ^]^ Electrochemical degradation of ether‐containing polymers was achieved by Stache and Lors using a catalytic amount of NHPI to yield monomeric products as well as molecular hydrogen (Scheme 6B).^[^ ^63^ ^]^ Recently, Luca designed a benzimidazolium organocatalyst (N‐DMBI) able to achieve single electron‐transfer to polyethylene‐terephthalate (PET), leading to depolymerization of PET and affording ethylene glycol and terephthalic acid with almost quantitative yields in a constant potential electrolysis (Scheme 6C).^[^ ^64^ ^]^ During the same year, a paired electrolysis strategy allowed a chlorination of electron‐rich arenes, using PVC as chlorinating source. The electro‐dechlorination of PVC was mediated by di(2‐ethylhexyl)phthalate (DEHP), a common PVC plasticizer. Hence the reaction proceeded efficiently with the direct use of PVC wastes and was selective toward the dechlorination of PVC. However, degradation was not complete and large polymer chains remained after electrolysis (Scheme 6D).^[^ ^65^ ^]^

### 3.3 Metallaelectro‐Catalyzed Degradation of Plastic

**Scheme 7:**
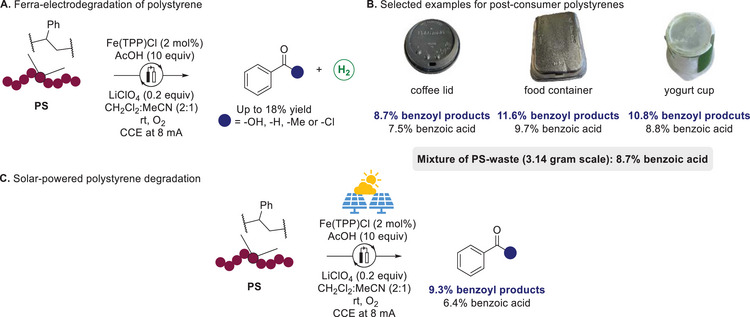
Iron‐catalyzed electrodegradation of polystyrene.

In 2024, an iron‐electrocatalytic re‐cycling of PS was accomplished by Ackermann through electro‐oxidative C–H functionalization (**Scheme** **7**A).^[^ ^66^ ^]^ The oxidative degradation reaction yielded up to 18% of benzoyl products in which benzoic acid represented the main degradation product, as well as small PS oligomers with less than 1% molecular weight retention compared to the parent polymer. The cathodic proton reduction for HER also exhibits interesting potential for a green hydrogen production. Moreover, this strategy was viable on gram‐scale and could be applied to diverse post‐consumer PS wastes, highlighting an outstanding tolerance toward dyes and additives (Scheme 7B). The iron‐electrocatalytic re‐cycling of PS was achieved using a commercial photovoltaic cell as energy source, thus demonstrating its potential for the conversion of renewable energy into benzoyl monomeric units (Scheme 7C).

**Scheme 8:**
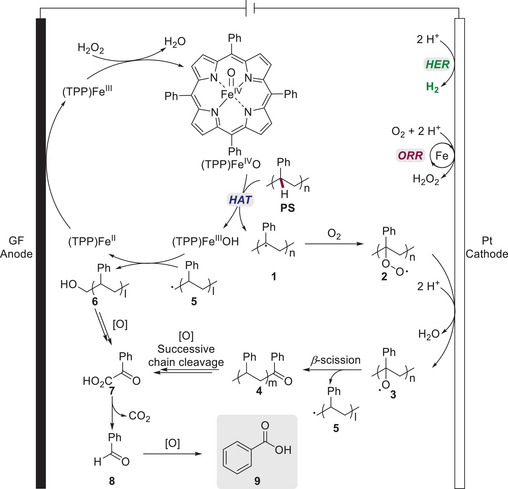
Mechanistic proposal for the iron‐electrodegradation of polystyrene.

Detailed mechanistic investigations provided insights into the mode of action of the electrochemical iron‐catalyzed PS‐degradation (**Scheme** **8**). According to Ackermann, the process is initiated by the oxidation of (TPP)Fe(III) with hydrogen peroxide, which is generated in situ via a cathodic iron‐mediated oxygen reduction reaction (ORR). The obtained (TPP)Fe(IV)O species generates the key radical **1** from PS through hydrogen atom transfer (HAT) from the polymer backbone with the concomitant formation of (TPP)Fe(III)OH. Radical **1** reacts with molecular oxygen to give intermediate **2,** which subsequently undergoes cathodic reduction to form the *O*‐centered radical **3**. Degradation of the latter through *β*‐scission of the polymer chain gives rise to species **4** and **5**. Afterwards, the oxidative chain cleavage of oligomer **4** results in the formation of phenyl‐glyoxylic acid **7**, which can undergo decarboxylation to form benzaldehyde **8** as a transient species. Ultimately, in a further oxidative process, benzoic acid **9** is formed as the main degradation product. The regeneration of the catalyst is accomplished through the radical rebound of **5** and subsequent anodic oxidation (Scheme 8).

Ultimately, the use of inexpensive, earth‐abundant, and non‐toxic catalyst combined with safe and scalable electrocatalysis shows a unique potential for tackling polymer re‐cycling, hence representing an attractive strategy for the development of future re‐cycling methods.^[^ ^67^ ^]^

## 4 Future Directions and Perspectives

During the last years, electrochemical organic chemistry has surfaced as a powerful strategy to perform polymer up‐ and re‐cycling. Electrochemistry offers a mild, cost‐efficient, and easily scalable alternative to traditional chemical transformations. Future opportunities in this rapidly emerging field include the selective and mild grafting of various functional groups into inert polyolefins backbones, as well as the development of efficient depolymerization reactions of different widely used and low recycled post‐consumer plastics such as PE, PP, or PVC.

Thus, the development of economical, efficient and scalable electrochemical degradation of commodity polymers is in high demand to address the major current challenge of chemical recycling of polymers. The carbon footprint and environmental impact of such methods needs to be quantified and minimized to allow further utilization and application of electrochemical polymer recycling. The scalability of electrochemical re‐cycling strategies needs to be further improved. In this context, flow electrosynthesis represents an exciting approach for future developments. Finally, merging organic electrochemistry with data sciences and machine learning technologies could tackle multidimensional problems of electrochemical systems in an efficient manner.^[^ ^68^, ^69^ ^]^

## Conflict of Interest

The authors declare no conflict of interest.
